# Effects of traditional Chinese medicine collaborative model (TCMCM) combined with adjuvant chemotherapy on IIIb and IIIc gastric cancer: a protocol for a randomized controlled trial

**DOI:** 10.1186/s13063-022-06013-5

**Published:** 2022-01-21

**Authors:** Zhaoyan Li, Guangtao Zhang, Nida Cao, Jingjuan Xu, Jiahuan Dong, Jia Li, Xiaohong Zhu, Yan Xu, Chen Han, Rui Wang, Xiang Xia, Gang Zhao, Xiangkun Huan, Jin Fan, Aiguang Zhao

**Affiliations:** 1 Department of Oncology, Longhua Hospital, Shanghai University of Traditional Chinese Medicine (TCM), 725 Wanping Road, Shanghai, 200032 China; 2 Department of Integrated Traditional and Western Medicine, Jinling Hospital, School of Medicine, Nanjing University, Nanjing, 210002 China; 3 Department of General Surgery, Renji Hospital, Shanghai Jiaotong University School of Medicine, Shanghai, 200240 China; 4 Department of Digestive tumor surgery, Jiangsu Province Hospital of Chinese Medicine, Nanjing University of Chinese Medicine, Nanjing, 210029 China; 5 Department of Radiation Oncology, Fudan University Shanghai Cancer Center, Shanghai, 200032 China

**Keywords:** Gastric cancer, Traditional Chinese medicine collaborative model, Adjuvant chemotherapy, DFS, Randomized controlled trial

## Abstract

**Background:**

Metastasis and/or recurrence can decrease the survival time of gastric cancer patients undergoing radical operation. Among them, those with stage IIIb and IIIc are especially at a high risk of metastasis and recurrence. The traditional Chinese medicine collaborative model (TCMCM) has been used in the treatment of cancer; however, its effects have not been systematically evaluated. This study is designed to evaluate whether TCMCM can decrease adverse effects after chemotherapy and reduce the recurrence and metastasis of stage IIIb and IIIc gastric cancer.

**Methods/design:**

This prospective, multicenter, randomized, open-label trial will recruit 260 patients with stage IIIb and IIIc gastric cancer who undergo radical surgery for D2 lymphadenectomy. The patients will be randomly assigned to receive usual adjuvant chemotherapy and TCMCM (intervention group) in a 1:1 ratio. Patients in the intervention group will receive an oral traditional Chinese formula, auricular acupressure, and acupoint therapy. All participants will receive usual adjuvant chemotherapy. The primary outcome is a 3-year disease-free survival rate. Secondary outcomes include quality of life, side effects caused by chemotherapy, and safety-related measures. Assessments will be performed during the screening period, at 4 and 8 cycles after adjuvant chemotherapy, and 9, 12, 18, 24, 30, and 36 months after randomization. Adverse events will be recorded. In addition, biological samples will be collected for mechanism analysis.

**Discussion:**

This will be the first clinical trial to evaluate the effects of TCMCM on disease-free survival (DFS) and quality of life in patients with stage IIIb and IIIc gastric cancer. Our results may be used to standardize TCMCM. We will also perform a larger-scale clinical trial in the future.

**Trial registration:**

ClinicalTrials.govNCT03607656. Registered on 1 July 2018. The final protocol version is V1.1.

## Background

Gastric cancer is the second leading cause of cancer-related death throughout the world [1]. Asian cases make up more than half of the world’s total, and Chinese cases make up about 42% of them [2, 3]. Due to a lack of effective diagnostic and therapeutic strategies, more than 80% of gastric cancer patients in China have already progressed into advanced stages at the time of diagnosis and their 5-year survival rate remains quite low [4]. In addition, patients with stage IIIb and IIIc gastric cancer are especially at a high risk of metastasis and recurrence, with a much poor survival than those in earlier stages.

With the continuous improvement in postoperative chemotherapy, the DFS and overall survival (OS) of gastric cancer have been prolonged [5, 6]. Several clinical trials have verified the effects of adjuvant chemotherapy in the treatment of gastric cancer [7–9]. Chemotherapy has been the standard treatment for patients with advanced gastric cancer [10]. However, about 40–60% of patients with gastric cancer after resection may undergo recurrence or metastasis [11]. The toxicity of chemotherapy may cause other side effects, thus impairing the quality of life of patients [12]. Therefore, there is still an urgent need to explore new methods to improve the prognosis of patients after radical gastrectomy.

TCM has shown beneficial effects in the treatment of gastric cancer [13, 14]. Auricular acupressure and acupoint therapy are nonpharmacological and noninvasive therapy that can diminish the side effects of chemotherapy and improve the quality of life [15, 16]. However, because a TCM treatment is always individualized and needs frequent adjustments, it is difficult to design a rigorous and modern controlled trial for the traditional Chinese medicine collaborative model (TCMCM). Large-scale, randomized controlled trials, especially high-quality designed clinical trials, are rare to evaluate the efficacy of TCMCM, especially in the treatment of gastric cancer [16]. The objectives of this study are to (1) evaluate whether TCMCM can reduce the recurrence and metastasis of stage IIIb and IIIc gastric cancer and decrease adverse effects after chemotherapy and (2) explore TCM biomarkers for the prognosis of gastric cancer.

### Study setting

This study will identify patients with stage IIIb and IIIc gastric cancer who undergo curative D2 gastrostomy since June 2018 at three centers in China, including Longhua Hospital Affiliated to Shanghai University of TCM, Renji Hospital Affiliated to Shanghai Jiaotong University School of Medicine, and Jiangsu Province Hospital of Chinese Medicine Affiliated to Nanjing University of Chinese Medicine.

## Methods/design

### Study design

**Fig. 1:**
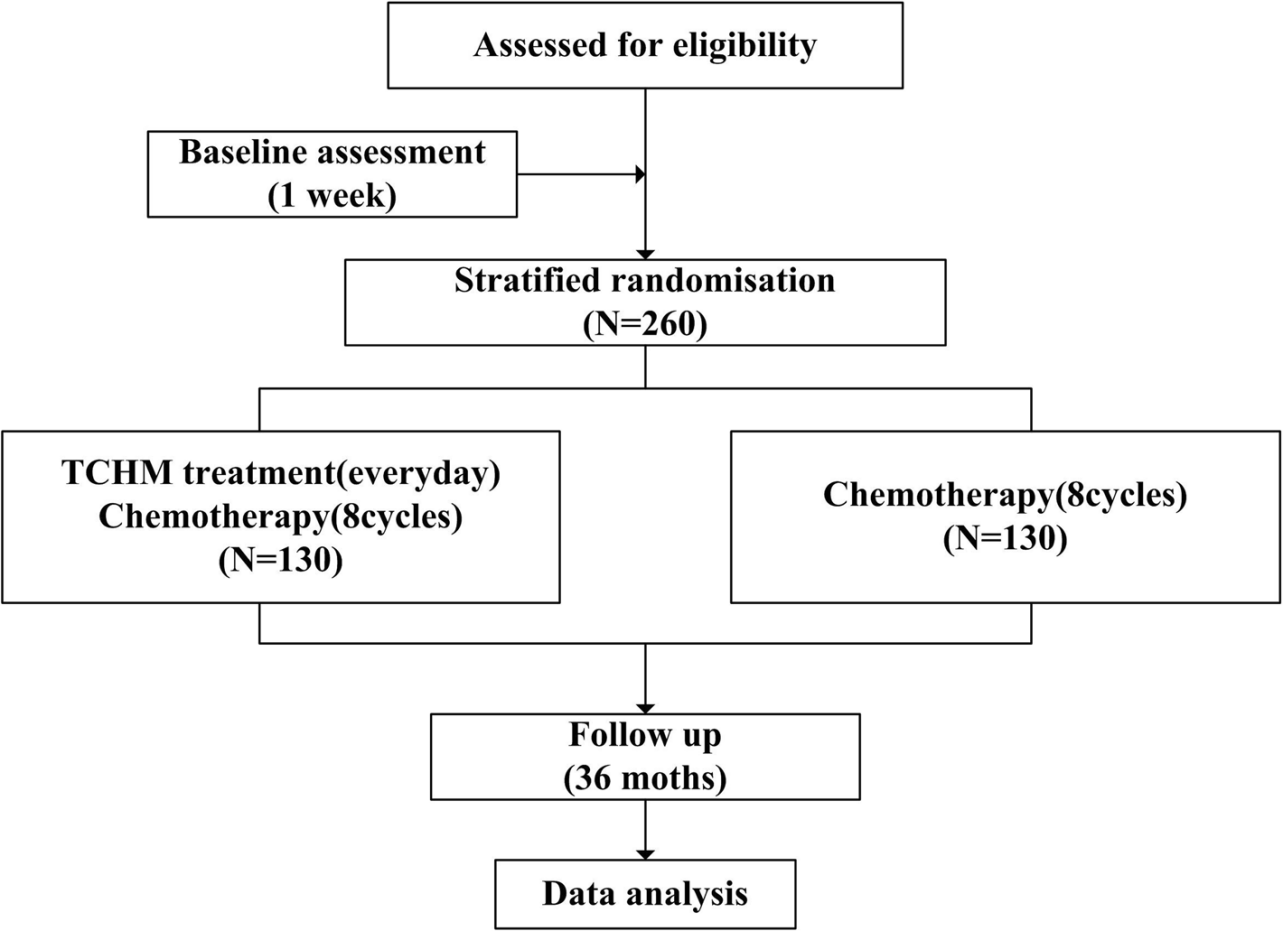
The trial design

This study protocol was registered in the ClinicalTrials Registry (https://register.clinicaltrials.gov/) on July 1, 2018 (No. NCT03607656). The present study is designed as a longitudinal randomized interventional study with two study arms. Patients in the intervention group will receive an oral traditional Chinese formula, auricular acupressure, and acupoint therapy. All participants will receive usual adjuvant chemotherapy continually, according to the National Comprehensive Cancer Network (NCCN) gastric cancer treatment guidelines. The trial design and checklist for this study protocol are summarized in Fig. 1 and Table 1, respectively.

**Table 1:** Schedule of enrollment and assessment

	Baseline (date)	Treatment period (cycle)								Follow-up (months)							
Time point	0	1	2	3	4	5	6	7	8	9	12	15	18	21	24	30	36
**Enrollment**																	
**Informed consent**	**√**																
**Eligibility screen**	**√**																
**General assessment**																	
**Disease data**	**√**																
**Family history**	**√**																
**Combined diseases**	**√**	**√**	**√**	**√**	**√**	**√**	**√**	**√**	**√**	**√**	**√**	**√**	**√**	**√**	**√**	**√**	**√**
**Medical examination**	**√**	**√**	**√**	**√**	**√**	**√**	**√**	**√**	**√**	**√**	**√**	**√**	**√**	**√**	**√**	**√**	**√**
**Combination therapy**	**√**	**√**	**√**	**√**	**√**	**√**	**√**	**√**	**√**	**√**	**√**	**√**	**√**	**√**	**√**	**√**	**√**
**Effectiveness observation**																	
**CT or MRI**	**√**				**√**				**√**	**√**	**√**		**√**		**√**	**√**	**√**
**Gastroscopy**											**√**				**√**		**√**
**Tumor markers**	**√**		**√**	**√**	**√**	**√**	**√**	**√**	**√**	**√**	**√**	**√**	**√**	**√**	**√**	**√**	**√**
**KPS score**	**√**		**√**	**√**	**√**	**√**	**√**	**√**	**√**	**√**	**√**	**√**	**√**	**√**	**√**	**√**	**√**
**QLQ-C30**	**√**				**√**				**√**	**√**	**√**	**√**	**√**	**√**	**√**	**√**	**√**
**DFS**			**√**	**√**	**√**	**√**	**√**	**√**	**√**	**√**	**√**	**√**	**√**	**√**	**√**	**√**	**√**
**Safety observation**																	
**Toxicity events**		**√**	**√**	**√**	**√**	**√**	**√**	**√**	**√**	**√**	**√**	**√**	**√**	**√**	**√**	**√**	**√**
**Adverse event**		**√**	**√**	**√**	**√**	**√**	**√**	**√**	**√**	**√**	**√**	**√**	**√**	**√**	**√**	**√**	**√**
**Data evaluation**																	**√**
**Audit medical records**																	**√**
**Ombudsman reviews medical records**																	**√**
**Outcome’s measurement**																	**√**

*CT* computed tomography, *MRI* magnetic resonance imaging, *KPS* Karnofsky Performance Status, *QLQ-30* Quality of Life Questionnaire-30, *DFS* disease-free survival

### Sample size estimation

Our previous study has described that the 3-year disease-free survival rate was 49% in patients receiving Chinese herbal medicine combined with chemotherapy and 28% in those receiving only chemotherapy. With a power of 0.9, the total number of randomized controlled trials is estimated as 197. Considering a follow-up loss rate of 20%, the confinement factor for the sample size will increase by 10% in different hospitals. Taken together, we will recruit a total of 260 patients in this trial.

### Recruitment

Before the research, the investigators of three centers will introduce this clinical trial to the patients and record their baseline data. Prospective participants will be informed of the benefit and possible risk associated with this study, so that they can withdraw from the trial at any time without specifying reasons. Eligible participants will be randomized into two groups with different treatments, once informed consent is obtained. The investigator must be qualified in conducting clinical trials. After qualification, the investigators are fixed. Every investigator will be trained to fully understand this clinical study.

### Criteria

#### Inclusion criteria

- Histologically confirmed gastric carcinoma treated with radical operation
- TNM (primary tumor, regional nodes, metastasis) stage IIIb or IIIc (the 8th edition of the tumor-node-metastasis staging system from the American Joint Cancer Committee/Union Internationale Contre le Cancer (AJCC/UICC))
•Onset age of 18 years old or above
- Karnofsky performance status higher than 70
- Life expectancy ≥ 6 months
- Adequate hepatic, renal, cardiac, and hematologic function
- Consent and compliment to long-term follow-up

#### Exclusion criteria

- Gastrectomy beyond D2, or TNM stage except for IIIb and IIIc
- Histological type except for gastric adenocarcinoma
- Conversion chemotherapy before surgery
- Concurrent cancer
- Women gravid or lactating
- Mental illness
- Uncontrolled significant comorbid conditions, such as unstable angina or myocardial infarction

#### Withdrawal criteria

- Patients can discontinue their participation in the study at any time for any reason without any consequences
- Those who have serious adverse events, complications, and special physiological changes, or if, in the investigator’s opinion, study medication should be stopped for any other reason

### Randomization

All eligible patients will be randomly assigned in a 1:1 ratio to the treatment or control group. In order to minimize selection bias, a computer-generated randomization sequence will be performed by a professional, independent statistician who is not involved in the recruitment process of the study. The central randomization software “Clinstat Group” APP (version 1.2.0) will be used for group allocation according to a computer-generated randomization number. Randomization is based on two stratification factors. The first one is the disease stage of IIIb or IIIc, and the second one is the center (Longhua Hospital, Renji Hospital, or Jiangsu Province Hospital of Chinese Medicine). Once the inclusion criteria are fulfilled and informed consent is received, the clinical researcher of centers will tell the statistician the information of patients’ gender, age, and TNM stage. When the statistician logs on the Clinstat Group to check this basic information, the result is immediately presented on the screen. Allocation concealment is guaranteed by the randomization program. The investigators are blind to the random numbers before the group allotment is revealed. Neither investigators nor patients are masked to treatment assignment in this open-label trial.

### Intervention

The patients in the control group will accept adjuvant chemotherapy. The chemotherapy is based on intravenous oxaliplatin 130 mg/m (2) on day 1 plus oral capecitabine 1000 mg/m (2) twice daily on days 1–14, every 21 days. Two additional chemotherapy regimens will be added after 27 June 2019, including intravenous oxaliplatin 100 mg/m (2) on day 1 plus oral S-1 40mg/m (2) twice daily on days 1–14, every 21 days; or intravenous docetaxel 40mg/m (2) on day 1 plus oral S-1 40mg/m (2) twice daily on days 1–14, every 21 days; or intravenous docetaxel 50mg/m (2) on day 1 plus intravenous oxaliplatin 85 mg/m (2) on day 1 plus 5-FU intravenous CIV 24h on day 1, every 14 days. Each participant will take eight cycles of chemotherapy. The chemotherapy is directed by one of the two chief physicians.

In addition to the above-mentioned adjuvant chemotherapy, patients in the treatment group will receive an oral traditional Chinese formula based on the Jianpi theory. The major components in the herbal formula include Radix Pseudostellariae (12g), Rhizoma Atractylodis Macrocephalae (12g), Poria (30g), Rhizome Pinelliae Preparata (9g), green tangerine peel (4.5g), Concha Ostreae (30g), and Prunella vulgaris (9g). These herbal components are provided by Sunbow Pharmaceutical (7600 Zhongchun Road, Shanghai 201100, China) (certified GMP ShanghaiG0172). The preparation of the formula water decoction and its quality control have been described in our previously published articles. The patient will take the oral decoction within 8–12 weeks after enrollment (one dose/day), and each dose is taken up in 2 to 4 times. The use of oral traditional Chinese formula is designed and adjusted by senior TCM physicians in every center, according to symptoms evaluated by examination, listening, smelling, interrogation, pulse-taking, and palpation. According to the clinical symptoms, the medication will be adjusted every 1~2 weeks, continue for at least 6 months, or until disease progression (recurrence/metastasis/die), unacceptable toxicity, or withdrawal of consent.

On the first day of every cycle of adjuvant chemotherapy, the patients will receive auricular acupressure and acupoint therapy performed by the research nurse. Auricular acupressure and acupoints points are chosen by a panel of expert faculty from Shanghai University of TCM. The auricular acupressure is performed at Shenmen, Jiaogan, and stomach. Magnetic beads are placed by a tape to stimulate these three auricular acupoints. One side of the ear is treated each time, and both sides of the ear are treated in turn (3 times per day, 3 min per time, 9 min in total). The acupoints selected for this study include Neiguan, Zusanli, and Fenglong, which will be treated for 24 h.

### Study implementation

Prior to implementation, the final protocol will be shown to researchers through pre-clinical trial training to inform them of plan goals, work flow, and methodology. The clinicians will timely evaluate the risks related to chemotherapy, closely monitor the adverse events, and adjust the dose of chemotherapy or stop it according to clinical needs. Researchers will record all the data during the treatment and follow-up period. Any problem will be reported to the investigator and resolved. The electronic report forms will be filled.

### Biological specimens

Next-generation sequencing (NGS) technology is used to observe the mutations of tumorigenesis-related genes and potential treatment-related genes in some patients and to assess the relationship between tumor mutation burden (TMB) and the prognosis of gastric cancer. Blood is collected at 3 time points: before surgery, after chemotherapy, and recurrence and metastasis. Patients without recurrence/metastasis will be examined for 24 months after enrollment or till the end of the study.

### Study outcomes

#### Primary outcome

The primary endpoint is a 3-year disease-free survival rate. DFS is defined as the date of randomization until the date of progression (tumor metastasis or recurrence) or the date of death from any cause. If symptoms or signs of suspected recurrence or metastasis or abnormal tumor markers are found during follow-up, CT/MRI/gastroscopy (PET-CT if necessary) should be performed promptly. The Response Evaluation Criteria in Solid Tumors version 1.1 (RECIST 1.1) will be used to estimate the objective curative effect. If a mass contains abnormal tumor markers, but normal CT or MRI results, it will be evaluated by a team of experts. Kaplan–Meier curves will be performed to evaluate intention-to-treat (ITT) and per-protocol (PP) at 2 years after the end of the study.

#### Secondary outcomes

##### EORTC QLQ-C30 scale

The European Organization for Research on Treatment of Cancer Quality of Life Questionnaire-Core 30 (EORTC QLQ-C30) has shown acceptable reliability in assessing the quality of life of cancer patients. All results are recorded in the CRF table and the efficacy is scored. Assessments will be performed during the screening period, after 4 and 8 cycles of adjuvant chemotherapy, and at 9, 12, 18, 24, 30, and 36 months after randomization.

#### Side effects of chemotherapy

Side effects during chemotherapy will be documented. NCI-CTC standard will be used to record adverse effects of treatments. Toxicity and adverse events are compared according to the NCI-CTC anti-cancer drugs in acute and subacute toxicity criteria and the Levi special sensory nerve toxicity grading criteria for oxaliplatin. Clinical researchers adjust medications based on toxic side effects. After the completion of treatment, an imaging examination will be conducted during the follow-up. When a dangerous or life-threatening adverse event occurs, the dose will be reduced or discontinued.

#### Safety

Safety will be assessed according to vital signs at each visit, weight, KPS score, blood, liver and kidney function, fecal routine, and electrocardiogram. Adverse events are defined as all unexpected or unintended symptoms, signs, or disorders, with no regard to their causality with chemotherapy. According to the NCI common adverse events evaluation standard (v4.02) to compare adverse events, if serious adverse events occur during the study period, the patient can withdraw from the study and needs to complete the examinations and evaluations as many as possible.

#### Follow-up

Tumor marker examinations are checked every 3 months during the first 2 years, then every 6 months. A full abdominal augmentation CT/MRI and chest CT is performed every 3 months in a year and then every 6 months; the gastroscope is examined once a year. All patients are followed up for 3 years or until recurrence, metastasis, or death.

### Data collection and management

The investigators and research coordinators will study the instructions for data collection before the trial begins. Only original medical records are collected and cannot be changed. The original record should not show any correction and can only be accompanied by an explanation, date, and physician signature. All patient data collected during this trial will be stored in an electronic case report form (eCRF).

### Data monitoring

The data will be analyzed according to the study protocol. Data administrators create accounts according to different identities, such as researchers, inspectors, and auditors, and grant permissions to EDC. Inspectors check the data on EDC and ask questions online at any time when they find problems. Researchers answer these questions and modify incorrect data. After verification by the inspector, the data of one patient will be locked by the manager, until the data of the last subject is locked.

### Auditing

The quality of documents, electronic case report form (eCRF), and follow-up records will be audited. Data monitoring will also be conducted by way of site visits. The Data Monitoring Committee (DMC) will be established by Xiyuan Hospital of China Academy of Chinese Medical Sciences. The data management does not involve clinical observation.

### Statistical analysis

SPSS Statistics version 22.0 will be used for data management and statistical analyses. All patients will be followed according to the protocol, and both intention-to-treat and per-protocol analyses will be performed. Missing data will be replaced by the information at the last observation to obtain a complete database. Independent sample *T* test and chi-square test (*χ*^2^ test) will be used to compare numerical variables and categorical variables between groups, respectively. When the distribution of variables is abnormal, a non-parametric test will be selected. Univariate analysis of survival is performed using the Kaplan–Meier method, and the log-rank test is used to compare the factors. The association of DFS with each clinical/demographic factor is evaluated using the Kaplan–Meier analysis. Multivariate data are analyzed using Cox regression analysis, trend testing, and so on. Statistical analysis will be carried out by Fudan University Cancer Hospital. Statistical analysts are not involved in clinical observations.

### Ancillary and post-trial care

Our team will provide one-to-one health care to each patient. Researchers regularly communicate with patients and observe their clinical manifestations. Moreover, we will provide psychological counseling to prevent the patient’s drop-off due to psychological disorders.

### Dissemination policy

All results from the study will be submitted for publication in international scientific journals. Furthermore, the papers will be communicated to clinical, scientific, and patients through presentations at international clinical and scientific conferences.

## Discussion

Clinical studies have demonstrated that TCM can improve the quality of life, reduce side effects [17], and prolong the overall survival of cancer patients [18, 19]. TCM treatment includes herbal medicine, acupuncture, acupoint therapy, and others. The herbal formula in this study was first designed in the 1980s. Our previous clinical study has shown that it can prolong the OS and DFS of patients with advanced gastric cancer [20, 21]. However, most of these studies are non-randomized observational methods that may carry a high risk of bias. The administration of TCM varies with a physician’s experience. Therefore, in the present study, we will use randomized controlled trials without blind methods to strictly control the biases. We will balance the confounding factors to objectively evaluate the value of TCMCM intervention in the treatment of stage IIIB and IIIC gastric cancer. In this study, the procedures of auricular and acupoint therapies are the same for the patients in the treatment group. Furthermore, free acupuncture and acupoint therapy are more acceptable and conducive to reducing the dropout rate. In addition, our team will provide one-to-one health care to each patient. Therefore, we believe that most patients should be able to cooperate with the treatment and ensure a good adherence to the program during the 2-year follow-up period.

In our randomized clinical trial, we will assess not only disease-free survival but also quality of life, side effects, and safety indicators. Side effects of chemotherapy affect prognoses in patients with gastric cancer. However, neither preventive nor therapeutic methods have been established for these side effects. The present study is the first multicenter, randomized, open-label study to clarify the effects of TCMCM on the survival and quality of life in patients with stage IIIB and IIIC gastric cancer after surgery.

To ensure its quality and outcomes, all the experiments will be performed under strict quality control. Our findings will provide an example to integrate TCM and Western medicine in the research of gastric cancer. TCMCM not only reduces the recurrence and metastasis rate of stage IIIb and IIIc gastric cancer, but also decreases adverse effects after chemotherapy. The small sample size is a limitation in this study. Nevertheless, the results will provide novel evidence about the effectiveness of adjuvant TCM in patients with stage IIIB and IIIC gastric cancer.

Our findings will provide an evidence-based evidence about the efficacy of TCMCM, which will open new insight into the clinical management of gastric cancer.

## Trial status

Protocol version: 4. Trial registration: ClinicalTrials.gov. Registration number: NCT03607656, https://clinicaltrials.gov/ct2/show/NCT03607656. Date of trial registration: 1 July 2018

Was this trial prospectively registered? No

Date recruitment began: June 2018. Recruiting is ongoing

Anticipated completion date: December 2021

## Data Availability

The datasets used during the current study are available from the corresponding author on reasonable request.

## Abbreviations

TCHM: Traditional Chinese herbal medicine
CT: Randomized control trial
DFS: Disease-free survival
AJCC/UICC: The American Joint Cancer Committee/Union Internationale Contre le Cancer
NCCN: National Comprehensive Cancer Network
IT: Intention-to-treat
PP: Per-protocol
EORTC QLQ-C30: European Organization for Research and Treatment of Cancer (EORTC) Gastric-Cancer-Specific Quality of Life Questionnaire
DMC: Data Monitoring Committee
NGS: Next-generation sequencing
TMB: Tumor mutation burden
